# Supplementary material for: Single-Domain Antibodies as Antibody–Drug Conjugates: From Promise to Practice—A Systematic Review
Source: Cancers (Basel). 2024 Jul 27;16(15):2681. doi: 10.3390/cancers16152681 (PMC11311928; doi:10.3390/cancers16152681)
Supplement: Supplementary file 1 [file cancers-16-02681-s001.zip › cancers-3101143-supplementary.pdf]

Supplementary

Table S1. Major differences between mAbs, VHHs and VHH-Fcs.

	mAb	***VHH***	***VHH-Fc***
Size	~14.5 nm	2.5 to 4 nm	~6.5 nm
MW	150 kDa	12-17 kDa	80-90 kDa
Antibody production	Mammalian cell post-translational modification needed	Mammalian or microbial, naked and no post-translational modification needed	Mammalian, includes Fc, may need modification
Immunogenicity and complexity	High, glycosylation and interactions with immune cells via Fc/FcR	Low, no Fc/FcR interaction	Moderate, includes Fc/FcR interactions
Stability	pH and temperature dependent and aggregation with other protein	High, even > 60 °C, low aggregation	High, similar to VHH but with longer half-life
Clearence	Hepatic, long half-life	Renal, relative short half live	Mixed (hepatic and renal), intermediate half-life
Tissue penetration	Low	High tissue permeability, can cross the BBB	Moderate, better than mAb but less than VHH
Epitope recognition	Difficult recognition hidden sites	Strong and site that cannot be reached for normal antibodies	Similar to VHH, with the advantage of longer half-life
Production cost and standarization	High	Relative low	Moderate, more costly than VHH but easier to produce than mAb
Humanization and structural modification	Can lost the function or stability	Easy modification	Easy modification with improved stability
Affinity	nM-μM	pM-nM	pM-nM
